# Supplementary material for: MetaRibo-Seq measures translation in microbiomes
Source: Nat Commun. 2020 Jun 29;11:3268. doi: 10.1038/s41467-020-17081-z (PMC7324362; doi:10.1038/s41467-020-17081-z)
Supplement: Supplementary file 10 — Supplementary Data 7 [file 41467_2020_17081_MOESM10_ESM.zip › File2/Confidence_High_Taxonomy/35890_out.krona.html]

Javascript must be enabled to view this page.

members
magnitude
magnitudeUnassigned
count
unassigned
taxon
rank

35890\_out

3

superkingdom
3
2

phylum
3
1239

186801
3
class

order
3
186802

1
family
31979

1485
1
genus

59620

SRS016335\_contig\_number\_contig-100\_308.243504
1
species

186803
family
1

1
genus
33042


SRS064232\_contig\_number\_3469
410072
species
1

541000
1
family

genus
1
1935176

1871018

SRS078419\_contig\_number\_28885
1
species
